# Motor Impairment Is the Key Determinant of the 1-Year Modified Rankin Scale Score in Intracerebral Haemorrhage

**DOI:** 10.1159/ced/accag008

**Published:** 2026-08-17

**Authors:** Elbert Edy, Ulrike Hammerbeck, David A. Jenkins, Daniel F. Hanley, Adrian R. Parry-Jones

**Affiliations:** a Foundation Programme, Manchester University NHS Foundation Trust, Manchester, UK; b Division of Cardiovascular Sciences, Faculty of Biology, Medicine and Health, University of Manchester, Manchester, UK; c Centre for Human and Applied Physiological Sciences, Faculty for Life Sciences and Medicine, King’s College London, London, UK; d Division of Informatics, Imaging and Data Science, Faculty of Biology, Medicine and Health, University of Manchester, Manchester, UK; e Department of Neurology, Division of Brain Injury Outcomes, John Hopkins University School of Medicine, Baltimore, MD, USA; f Geoffrey Jefferson Brain Research Centre, Manchester Academic Health Science Centre, Northern Care Alliance and University of Manchester, Manchester, UK

**Keywords:** Intracerebral haemorrhage, Neurological impairments, Functional outcome, Modified Rankin Scale

## Abstract

**Introduction:**

Intracerebral haemorrhage (ICH) is associated with significant long-term disability. Precise ICH location is readily available from routine diagnostic imaging and may thus enhance prognostic models. We have previously shown that involvement of motor pathways in the haematoma is highly predictive of poor outcome. However, ICH patients suffer many other impairments, and this is largely governed by ICH location. To better understand the potential prognostic significance of ICH location and guide further research, our aim was to investigate the associations between specific domains of neurological impairment and 1-year functional outcome after ICH and how these association change over time.

**Methods:**

Data were pooled from 2 ICH trials: CLEAR-III and MISTIE-III. National Institutes of Health Stroke Scale and Stroke Impact Scale-3 subscores from day 30, 180, and 365 were used to construct eight neurological domains: consciousness, language, motor, sensory, vision, ataxia, memory and thinking, and emotions. The primary outcome was 1-year mRS in ICH survivors, dichotomised into good (0–3) or poor (4–5) outcome. Multivariable logistic regression models were used to investigate the association between impairments at days 30, 180, and 365 and good 1-year mRS, adjusting for baseline confounders (age, premorbid mRS, ICH, and intraventricular volume on stability CT scans) and treatment group (intervention vs. control).

**Results:**

A total of 736 patients were included in the analysis. Motor (OR 0.937; 95% CI: 0.925–0.950) and vision (OR 0.991; 95% CI: 0.983–0.999) impairment at day 30 were associated with poor 1-year mRS. At day 180, motor (OR 0.891; 95% CI: 0.874–0.908) and memory and thinking (OR 0.987; 95% CI: 0.975–0.998) impairment were associated with poor 1-year mRS. At day 365, motor (OR 0.876; 95% CI: 0.856–0.897) impairment was significantly associated with poor 1-year mRS, along with memory and thinking (OR 0.982; 95% CI: 0.971–0.994) and language (OR 0.982; 95% CI: 0.966–0.999) impairment.

**Conclusion:**

In our secondary analysis of this ICH cohort, motor impairment within the first year after ICH is consistently and strongly associated with the 1-year mRS at all timepoints. Although the associations are weaker, memory and thinking at days 180 and 365 was also associated with 1-year mRS. Among the other domains, only vision showed a weak association at a single time point (day 30). Our exploratory study suggests that lesion locations impacting on motor function and memory and thinking may worsen functional recovery in ICH patients.

## Introduction

Although spontaneous intracerebral haemorrhage (ICH) constituted 28.8% of all new strokes in 2021, it accounted for nearly half of stroke-related mortality (45.6%) and has the highest age-standardised disability-adjusted life-years rate [1]. A wide range of factors have been identified as predictors of post-ICH disability, including premorbid dependence, ICH volume, and location [2]. Haemorrhage location is typically classified as lobar vs. deep, and supratentorial vs. infratentorial [3]. Deep and infratentorial ICH locations are associated with poor outcomes [2, 4], but this classification lacks granularity and may overlook valuable prognostic information. Using both manual segmentation and a machine learning approach to predict the location of the corticospinal tract on baseline CT brain scans, we have shown that motor pathway involvement in ICH on baseline CT, is a strong and independent prognostic factor [5, 6]. However, ICH survivors typically suffer a range of neurological deficits beyond motor impairment. If other impairments are equally predictive of poor outcome, locations which drive these impairments may also be of significant prognostic value. Previous studies looking at the impact of impairments on disability have not been conducted in an exclusively ICH cohort [7], and few have assessed a wide range of impairments concurrently [8–11]. In this secondary analysis of data from two large ICH randomised controlled trials (RCTs), we used standardised and routinely collected data to capture impairments in ICH survivors at multiple timepoints and investigate their associations with the 1-year modified Rankin Scale (mRS).

## Methods

### Sources of Datasets and Patients

We conducted a secondary analysis using data from two RCTs: (1) Clot Lysis: Evaluating Accelerated Resolution of Intraventricular Haemorrhage (CLEAR-III) trial (ClinicalTrials.gov, NCT00784134) [12]; (2) Minimally Invasive Surgery plus Alteplase for Intracerebral Haemorrhage Evacuation (MISTIE-III) (ClinicalTrials.gov, NCT01827046) [13]. CLEAR-III recruited intraventricular haemorrhage (IVH) patients with obstructive hydrocephalus, an external ventricular drain (EVD), and a small-moderate ICH (<30 mL) and randomised them to IVH removal with alteplase vs. 0.9% saline via the EVD. MISTIE-III enrolled patients with large ICH (>30 mL) who were randomised to either receive haematoma removal using minimally invasive surgery followed by alteplase, or standard medical treatment. We excluded patients who died by day 365 or had missing 1-year mRS data.

### Measurements and Outcome

Baseline characteristics included age, sex, race, premorbid mRS, Glasgow Coma Scale (GCS), National Institutes of Health Stroke Scale (NIHSS) score, and ICH and IVH volume on stability CT scans. NIHSS and the Stroke Impact Scale-3 (SIS-3) scores from days 30, 180, and 365 were used to measure neurological impairments.

NIHSS items were categorised into six domains: consciousness, language, motor, sensory, vision, and ataxia [14]. We used SIS-3 scores from memory and thinking and emotions domains as surrogate measures of impairments as these domains are not evaluated by the NIHSS. Although not originally designed to measure impairments, a previous study has found that the memory and thinking and emotions domains from SIS-3 can detect cognitive deficits [15]. Overall, 8 domains were constructed from the NIHSS and SIS-3 components (Table 1).

**Table 1.** Domains of neurological impairment derived from subgroups of the National Institutes of Health Stroke Scale (NIHSS) and Stroke Impact Scale-3 (SIS-3)

Impairments	NIHSS items	Total raw score (range)
Consciousness^a^	1a + 2	5 (0–5)
Language^a^	1b + 1c + 9 + 10	9 (0–9)
Motor^a^	4 + 5a + 5b + 6a + 6b	19 (0–19)
Sensory^a^	8 + 11	4 (0–4)
Vision^a^	3	3 (0–3)
Ataxia^a^	7	2 (0–2)

Impairments	SIS-3 items	Total raw score
Memory and thinking^b^	2a + 2b + 2c + 2d + 2e + 2f + 2g	35 (1–35)
Emotions^b^	3a + 3b + 3c + 3d + 3e + 3f + 3g + 3h + 3i	45 (1–45)

^a^ Derived from NIHSS subgroups. Higher scores correlate with higher severity of impairment.

^b^ Derived from SIS-3 subgroups. Higher scores correlate with lower severity of impairment.

The total raw score for each SIS-3 domain is scaled to a range of 0–100 using the following formula [16]:

$$Scaled\;Score=\left[\frac{Actual\;Raw\;Score-Lowest\;Possible\;Raw\;Score}{Possible\;Raw\;Score\;Range}\right]*\;100$$

To facilitate comparisons between domains derived from the NIHSS and SIS-3, the same formula was applied to NIHSS scores. As higher SIS-3 scores indicate less impairment, values were reversed to align with NIHSS:

ReversedScaledSIS−3Score=100–OriginalScaledSIS−3Score

The primary outcome was 1-year mRS, which was centrally adjudicated by blinded assessors [17].

### Statistical Analysis

We compared baseline characteristics between the CLEAR-III and MISTIE-III trial using a *t* test or Mann-Whitney test for continuous, and Chi-square or Fisher’s exact test for categorical variables.

Patients were dichotomised into good (mRS 0–3) or poor (mRS 4–5) functional outcome, consistent with the original trials and other major ICH trials [18]. Three separate multivariable logistic regression analyses were performed to test the independent association between the severity of neurological deficits at days 30, 180, and 365, and good 1-year outcome. All eight scaled domain scores were entered simultaneously into each model, adjusting for age, premorbid mRS, stability ICH, IVH volume [2], and treatment group (intervention vs control). Baseline NIHSS and GCS were excluded to avoid potential collinearity with NIHSS-derived domains and baseline severity markers. Trial cohort was not included to avoid additional collinearity, as major differences between trials were already accounted for. Missing data for each timepoint (online suppl., Table 1; for all online suppl. material, see https://doi.org/10.1159/ced/accag008) were imputed independently of other timepoints using predictive mean matching method, generating 15 imputed datasets over 20 iterations. Logistic regression was performed on each imputed dataset, and estimates were pooled using Rubin’s rules to obtain final regression coefficients and standard errors. A post hoc analysis of multicollinearity was performed by calculating variance inflation factors for all predictors across the 15 imputed datasets.

Descriptive statistical analyses were performed using IBM SPSS Statistics for Mac Version 29.0 (IBM Corp., Armonk, NY, USA). Logistic regression and multiple imputation were performed using the “mice” package [19] in *R* version 4.4.0 (2024-04-24). Statistical significance was defined as *p* value of <0.05.

### Sensitivity Analysis

Four sensitivity analyses were conducted. Firstly, complete case analysis was performed, in which the primary analyses were performed without imputing missing values. Secondly, patients who died before 1 year (mRS = 6) were included in the poor functional outcome group to assess for survivor bias. Thirdly, for the day-180 model, interaction terms between day-30 and day-180 impairments were tested to evaluate whether the impact of later impairments depended on earlier severity, as studies suggest major recovery occurs within 4–6 months [20, 21]. Fourthly, we tested an alternative dichotomisation of the mRS, defining good mRS as 0–2.

## Results

Out of 1,006 patients from MISTIE-III and CLEAR-III, 11.5% died by day 30 (*n* = 116), rising to 24.7% (*n* = 248) by day 365 (online suppl. Fig. 1). MISTIE-III had a lower case-fatality than CLEAR-III at day 365 (21.9% vs. 27.4%, *p* = 0.047) (online suppl. Fig. 2). However, amongst the survivors, CLEAR-III had a higher proportion of patients (68.3%) who achieved a good mRS at day 365 than MISTIE-III (55.4%) (*p* <0.001) (Table 2). A total of 736 patients were included in the final analysis (353 from CLEAR-III, 383 from MISTIE-III), with 22 excluded due to missing mRS score at 1 year (Fig. 1).

**Table 2.** Baseline characteristics of CLEAR-III and MISTIE-III survivors at 1 year

	CLEAR-III survivors at 1 year (*n* = 353)	MISTIE-III survivors at 1 year (*n* = 383)	*p* value	Combined (*n* = 736)
Age, mean (SD)	58.8 (10.8)	60.4 (12.7)	0.073	59.6 (11.8)
Male sex (%)	198 (56.1)	238 (62.1)	0.095	436 (59.2)
Race (%)				
White	220 (62.3)	282 (73.6)	<0.001	502 (68.2)
Black	115 (32.6)	71 (18.5)		186 (25.3)
Other	15 (4.2)	30 (7.8)		45 (6.1)
Unknown	3 (0.8)	–		3 (0.4)
Premorbid mRS (%)				
0	309 (87.5)	360 (94.0)	0.002	669 (90.9)
1	44 (12.5)	23 (6.0)		67 (9.1)
GCS at randomisation, median (IQR)	10 (7–13)	10 (8.5–13)	<0.001	10 (8–13)
Total NIHSS score at randomisation, median (IQR)	18 (11–32)	19 (15–23)	0.927	19 (14–25)
Consciousness	2 (1–3)	2 (1–3)		2 (1–3)
Language	5 (3–8)	5 (2–7)		5 (3–7)
Motor	9.0 (5.0–17)	10 (8–12)		10 (7–13)
Sensory	1 (0–4)	2 (1.0–3)		2 (0–3)
Vision	0 (0–2)	1 (0–2)		0 (0–2)
Ataxia	0 (0–0)	0 (0–0)		0 (0–0)
Stability CT (last CT prior to enrolment)				
ICH volume (mL), median (IQR)	7.5 (2.4–15.0)	45.3 (35.7–58.2)	<0.001	27.3 (8.2–46.3)
IVH volume (mL), median (IQR)	21.1 (11.6–37.5)	0.4 (0.0–3.0)	<0.001	6.3 (0.3–21.2)
Presence of IVH on stability scan (%)				
Present	353 (100)	240 (62.7)	<0.001	593 (80.6)
Absent	0 (0)	143 (37.3)		353 (19.4%)
Treatment group				
Intervention (%)	170 (48.2)	194 (50.7)	0.547	364 (49.5)
Control (%)	183 (51.8)	189 (49.3)		372 (50.5)
mRS at 1 year				
mRS 0–3 (%)	241 (68.3)	212 (55.4)	<0.001	453 (61.5)
mRS 4–5 (%)	112 (31.7)	171 (44.6)		283 (38.5)

SD, standard deviation; mRS, modified Rankin Scale; GCS, Glasgow Coma Scale; IQR, interquartile range; NIHSS, National Institute of Health Stroke Scale; CT, computed tomography; ICH, intracerebral haemorrhage; ml, millimetres; IVH, intraventricular haemorrhage.

**Fig. 1.**
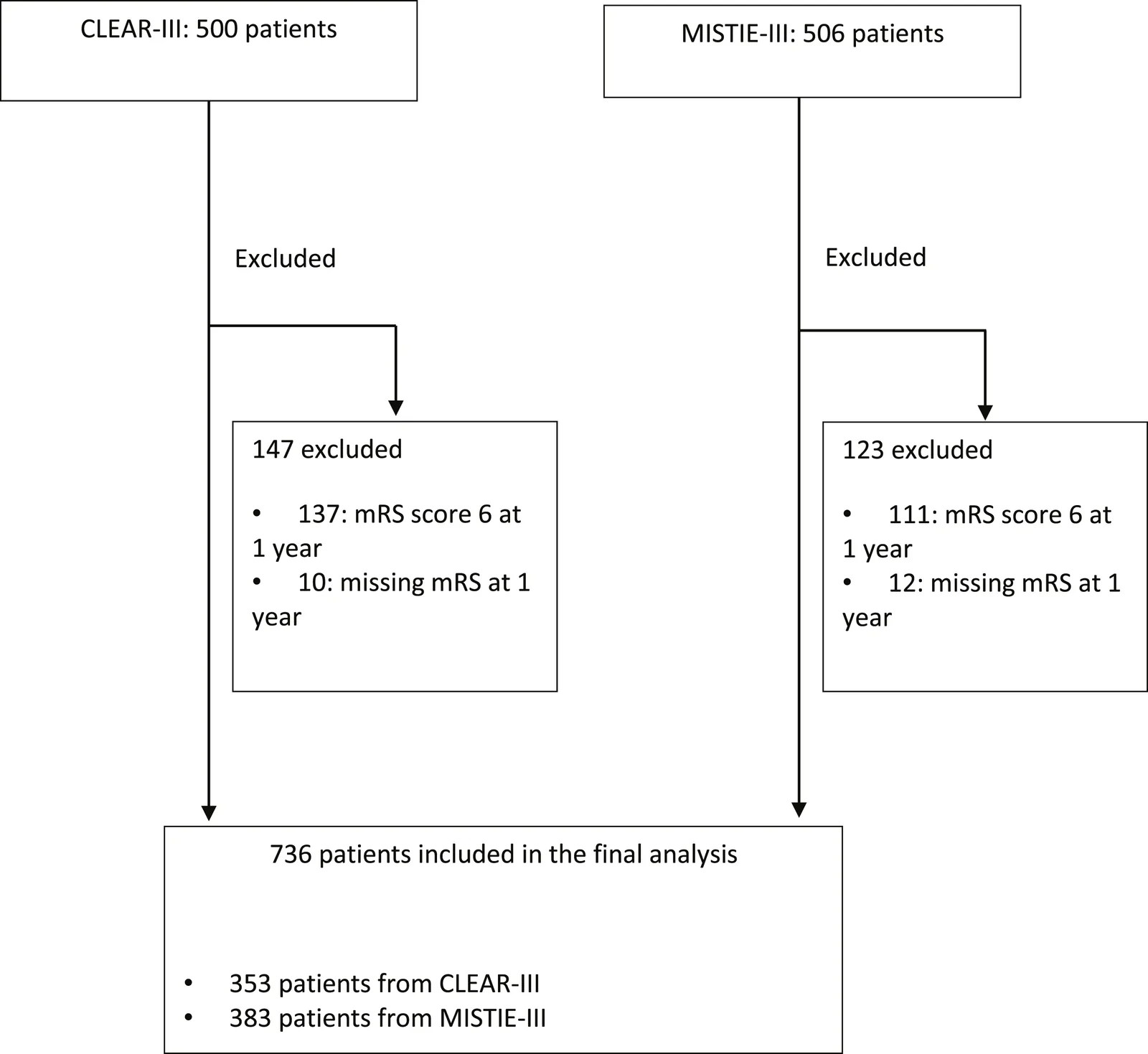
Flowchart of patients who were included in the analysis.

### Patient Demographics and Baseline Characteristics

Mean age was 59.6 years (SD 11.8), 59.2% were male, 68.2% were White, 25.3% were Black, and 6.1% were of other races. No statistically significant difference was found in baseline NIHSS scores between the two trial groups (*p* = 0.927) (Table 2). Expected differences in ICH and IVH volume and presence of IVH between the MISTIE-III and CLEAR-III cohorts based on inclusion criteria are evident on Table 2 [12, 13].

### Associations between Impairments at Different Timepoints (i.e., Day 30, 180, and 365) and 1-Year mRS Outcome

Motor impairment showed the strongest association with poor 1-year mRS across all timepoints. At day 30, motor (OR 0.937; 95% CI 0.925–0.950) and vision (OR 0.991; 95% CI 0.983–1.000) were associated with poor 1-year mRS. At day 180, memory and thinking impairment (OR 0.987; 95% CI 0.975–0.998) was also associated with poor 1-year mRS but to a lesser extent than motor (OR 0.891; 95% CI 0.874–0.908). One year after ICH, language impairment also demonstrated negative association with the 1-year mRS (OR 0.982; 95% CI 0.966–0.999) (Fig. 2). Baseline covariates were not significantly associated with 1-year mRS (online suppl. Fig. 3).

**Fig. 2.**
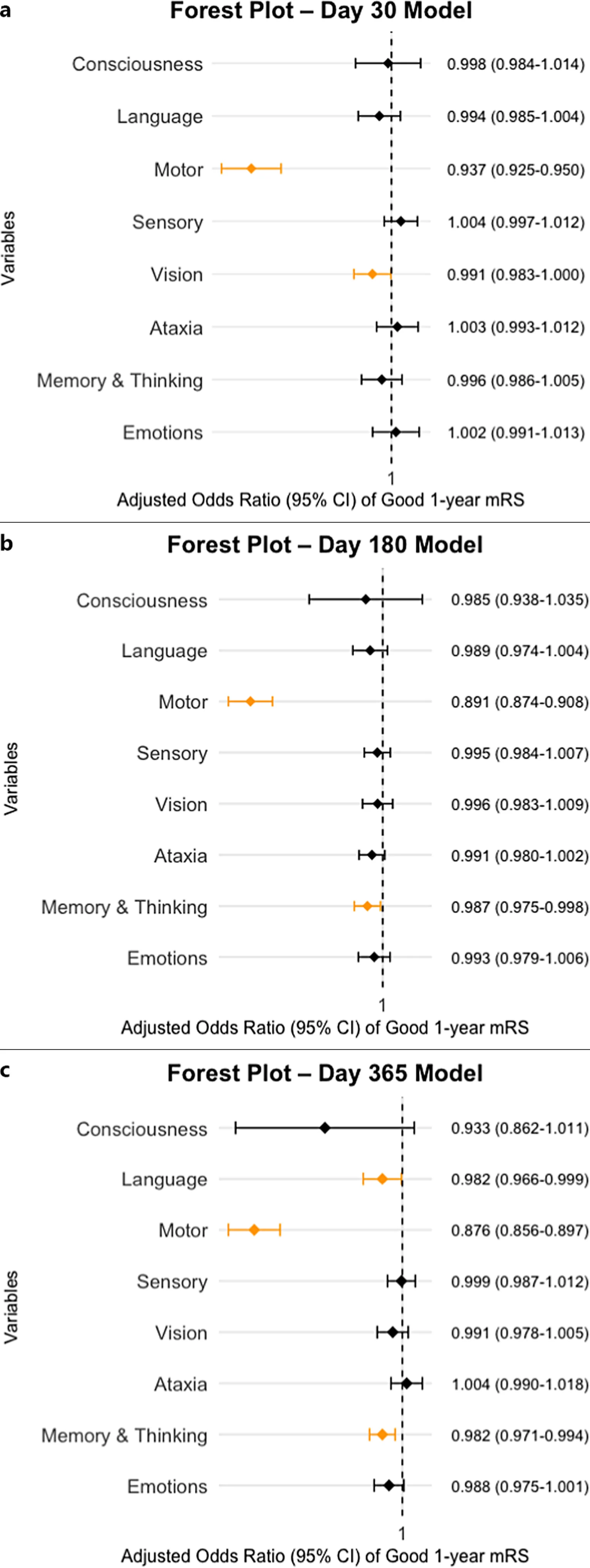
Forest plots of multivariable logistic regressions examining associations between neurological impairments and good modified Rankin Scale (mRS) at 1 year after intracerebral haemorrhage (ICH). Each panel displays adjusted odds ratios (ORs) and 95% confidence intervals (CIs) for impairments measured at day 30 (**a**), day 180 (**b**) , and day 365 (**c**), respectively. Outcome was defined as achieving mRS 0–3 at day 365. ORs <1 indicate greater impairment is associated with lower odds of good outcome. Significant associations (*p* < 0.05) are highlighted in orange. Models are adjusted for age, premorbid mRS, ICH, and intraventricular haemorrhage (IVH) volume on stability CT scan, and treatment group (not displayed on the plots).

Post hoc assessment of multicollinearity showed mean variance inflation factor values below 2.5 for all predictors across all three timepoint models (online suppl. Table 2). These findings indicate that multicollinearity is unlikely to have influenced the regression estimates.

### Sensitivity Analyses

In the complete case analysis, vision at day 30 was no longer associated with 1-year mRS while consciousness at day 365 became significant (online suppl. Fig. 4). Results were similar to the primary analysis when patients with mRS score of 6 were included (online suppl. Fig. 5). In the model assessing days 30 and 180 interactions, impaired motor function at day 30 and day 180 and sensory impairment at day 30 were associated with poor 1-year mRS. None of the interaction terms were significant (online suppl. Table 3). Impaired vision at day 30 and memory and thinking impairment at day 180 were no longer associated with poor 1-year mRS. When good outcome was redefined as mRS 0–2, previous associations in the primary analysis remained and more impairments, such as ataxia and emotions, became associated with outcome. In the model evaluating impairments at day 365, being in the intervention arm was also associated with poor 1-year outcome (online suppl. Fig. 6).

## Discussion

Our exploratory study found that in an exclusively ICH cohort, motor impairment at days 30, 180, and 365 had the strongest association with poor 1-year mRS. Of all other domains significantly associated with 1-year mRS, only memory and thinking were significant at more than one time point. This is consistent with a multicentre observational study of ischaemic stroke and ICH patients which demonstrated that motor impairment was the strongest predictor of poorer health-related quality of life (HRQoL) [22]. The increase in significantly associated impairments when using a different definition of good outcome (mRS 0–2) is likely attributable to key discriminators between mRS scores. A key discriminator between mRS scores of 3 and 4 is the ability to walk, whereas discrimination between scores of 2 and 3 depends on the participants ability to look after their own affairs without assistance. However, for both definitions of a good outcome, association with motor impairment remains highly significant.

Our study is novel as it focuses exclusively on an ICH cohort and evaluates a wide range of impairments. Previous studies investigating the effects of impairments on functional outcomes often used global measures (e.g., MMSE for cognition), have not assessed multiple domains concurrently or were not conducted in ICH-specific populations [8–11, 22]. Given the distinct pathophysiology in ICH versus ischaemic stroke, recovery trajectories are likely to differ. For instance, motor recovery is greater and occurs later in ICH, while language recovery tends to be greater in ischaemic stroke [23]. Hence, the prognostic utility of impairments may vary by stroke subtype. Evaluating multiple impairments concurrently helps identify those independently associated with outcome, while accounting for coexistent deficits. However, as this is an exploratory study, the observed associations should be considered as hypothesis generating.

Our findings demonstrate that the impairments associated with 1-year mRS differ across timepoints, which may reflect variations in recovery trajectories between neurological deficits. Studies on ischaemic stroke found variable degrees and rates of recovery for different deficits after thrombolysis [24, 25]. In ICH, a small observational study showed sensory and lower limb impairments levelled off after 3 months, while upper limb and ambulatory function improved up to 6 months [21]. If certain impairments recover more substantially over time, their severity at earlier timepoints may become less predictive of long-term functional disability, whereas impairments with slower or incomplete recovery may show stronger or more persistent associations with 1-year mRS. This may explain the stronger association observed for motor impairment in our study. However, the generalisability of the existing ICH literature is limited by small sample sizes and incomplete assessment of impairment domains. In addition, our study did not directly compare recovery trajectories between deficits.

A key strength of our study is the standardised, repeated and accurate measurements of impairment available from the trial data as well as the centrally adjudicated, blinded mRS scores. However, there are several limitations to our study. Firstly, the NIHSS and SIS-3 subdomains used to assess specific neurological domains have not been well validated. SIS-3 is intended to measure HRQoL rather than impairments. Secondly, the NIHSS-derived domains may capture impairments with varying sensitivity. For example, the motor domain has a total score of 19 points, while sensory impairment has only four points, giving motor greater granularity. Thirdly, the SIS-3 is patient-reported and may lack objectivity in measuring memory and emotion and likely contributed to substantial missing data at day 30 (online suppl. Table 1) as participants were unable to answer questions pertaining to their life outside of the hospital. Fourthly, the dichotomisation of mRS has often been criticised for its loss of granularity. Ordinal analysis of the mRS was not possible in our study due to limited sample size. Fifthly, we have used data from randomised clinical trials of surgical interventions so participants may not be representative of patients seen in routine care. Further, surgery may have impacted on our results despite the overall neutral results of both trials and our adjustment for treatment group. Finally, this is an exploratory study with multiple analyses performed without adjustment for multiplicity; therefore, our findings should be viewed as hypothesis generating.

In conclusion, in this secondary analysis of a large cohort of ICH survivors, motor impairment at days 30, 180, and 365 are strongly associated with poor 1-year mRS. Weaker but statistically significant associations were found for memory and thinking at two timepoints. Unlike in ischaemic stroke, lesion location is readily available on routine diagnostic CT brain scans. How ICH location interacts with brain structures and tracts may improve prognostication in acute ICH. Our findings suggest that regions associated with motor impairment, and to a lesser extent memory and thinking, may help refine prognostic models in ICH.

## Acknowledgments

We would like to thank the participants of CLEAR-III and MISTIE-III trials for their contribution to this research.

## Statement of Ethics

This study used the fully anonymised dataset from MISTIE-III and CLEAR-III. Ethical approval for MISTIE-III and CLEAR-III was obtained from the local Institutional Review Boards or Ethics Committees at all participating sites. The data were collected with participant consent for future data sharing. Therefore, ethical approval is not required for this study in accordance with local or national guidelines. Written informed consent was obtained from all subjects or their legally authorised representatives before enrolling to the trials.

## Conflict of Interest Statement

The authors have no conflicts of interest to declare.

## Funding Sources

A.PJ is supported by a Margaret Giffen Reader Award (Ref: SA L-RC 19\100000). MISTIE III trial was supported by a grant from the National Institutes of Health (NIH), National Institute of Neurological Disorders and Stroke (NIH/NINDS) (U01NS080824) and materials grants from Genentech. CLEAR-III was supported by a grant from the NIH/NINDS (5U01 NS062851-05), which was awarded to DFH.

## Author Contributions

A.P.J. and U.H. conceived the study idea and study design. E.E. performed data analysis. D.A.J. advised on data analysis. D.F.H. provided the trial data. E.E., U.H., and A.P.J. drafted the manuscript. All authors critically reviewed, edited and approved the final version of the manuscript prior to submission.

## Data Availability

Individual deidentified participant data from CLEAR-III and MISTIE-III may be obtained by submitting a formal proposal to the trial steering committees or to the National Institute of Neurological Disorders and Stroke trial repository. Further enquiries can be directed to the corresponding author.
